# COVID-19 Vaccine Hesitancy Among Deployed Personnel in a Joint Environment

**DOI:** 10.1093/milmed/usab518

**Published:** 2021-12-11

**Authors:** Jason D Higginson, Dmitry Tumin, Timothy C Kuehhas, Susan E DeLozier-Hooks, Carl A Powell, Dale D Ramirez, Anja Dabelić, Michael R Basso

**Affiliations:** Office of the Dean Brody School of Medicine, East Carolina University, Greenville, NC 27858, USA; Office of the Dean Brody School of Medicine, East Carolina University, Greenville, NC 27858, USA; United States Navy, USA; United States Army, USA; United States Navy, USA; United States Navy, USA; United States Navy, USA; Department of Psychiatry and Psychology, Mayo Clinic, Rochester, MN 55902, USA

## Abstract

**Introduction:**

In the United States, vaccine hesitancy has been identified as a major barrier to vaccination against COVID-19, but attitudes toward COVID-19 vaccination among military personnel are not well understood. We evaluated the prevalence and correlates of COVID-19 vaccine consent or refusal among deployed personnel in a joint environment.

**Materials and Methods:**

Deidentified data were retrospectively extracted from the electronic medical record of the Military Health System in May 2021. All personnel currently assigned to the deployment area of operations were included in the analysis if their choice to receive the vaccine was known. Personnel characteristics were compared by vaccine acceptance status using chi-square tests, Fisher’s exact tests, or correlation coefficients. This analysis was exempted from Institutional Review Board review.

**Results:**

The sample included 1,809 individuals, primarily members of the Army (72%) and members of Reserve (53%) or National Guard (27%) units. In the overall sample, 61% accepted the vaccine, with vaccine acceptance rates being lowest among Black or African American personnel (54%; *P* = .03 for comparison across racial groups) and members of Reserve or National Guard units (59%; *P* < .001 for comparison by component). No differences in vaccine acceptance were found according to sex or health status (including prior COVID-19 infection).

**Conclusions:**

Overall vaccine acceptance was greater among deployed military personnel than that reported in the U.S. population as a whole. However, lower vaccine acceptance among personnel from marginalized populations suggests a need to ensure that all service members have sufficient opportunities to have a frank and ongoing discussion with health care providers to address concerns related to vaccination. Additionally, lower vaccine acceptance among Reserve and National Guard personnel indicates a need for innovative educational approaches to counter vaccine hesitancy in the premobilization phase of deployment.

## INTRODUCTION

The emergence of the novel coronavirus disease in 2019 (COVID-19) presented a new threat to military force readiness.^1^ Whereas initial preventive actions centered on reducing viral transmission,^2,3^ the introduction of efficacious vaccines for COVID-19 in late 2020 and early 2021 has shifted the emphasis to vaccinating personnel against the SARS-CoV-2 virus.^4,5^ In the United States, vaccine hesitancy has been identified as a major barrier to COVID-19 vaccination in the civilian population.^6^ Attitudes toward COVID-19 vaccination among U.S. military personnel are not well understood. In this population, COVID-19 vaccine hesitancy may be related to younger age,^7^ racial and ethnic differences in vaccine acceptance,^5^ and uncertainty about the need for vaccination after recovering from COVID-19 infection.^8^ Understanding barriers to vaccine acceptance among military personnel is important for designing educational programs to increase vaccine uptake in this population. In this project, we evaluated the prevalence and correlates of COVID-19 vaccine consent or refusal among deployed personnel in a joint environment. We aimed to determine whether personnel who have recovered from COVID-19 before mobilization were less likely to consent to vaccination and whether vaccination consent varied according to demographic characteristics, military branch, and pre-existing health conditions.

## METHODS

This analysis was exempted from review by the Naval Medical Readiness and Training Command Portsmouth Institutional Review Board. Deidentified data were retrospectively extracted from the electronic medical record (EMR) of the Military Health System (MHS) in May 2021. Some data points were missing due to paper documentation not being scanned into the EMR. COVID-19 vaccinations were offered from January through May 2021. All personnel currently assigned to the deployment area of operations were evaluated for inclusion in the data analysis. Individuals for whom choice to receive the vaccine was missing were excluded.

The primary outcome was vaccine acceptance status. Additional variables queried from the MHS included age, gender, race and ethnicity (self-reported data entered in the military health record), military branch, body mass index (BMI), and health conditions considered to be high risk for COVID-19 infection. These health conditions were identified based on Centers for Disease Control and Prevention guidance and included chronic kidney disease; chronic obstructive pulmonary disease; obesity (BMI of 30 or higher); immunocompromised state from solid organ transplant; serious heart conditions, such as heart failure, coronary artery disease, or cardiomyopathy; sickle cell disease; and type 2 diabetes. Personnel were classified as to whether they had a qualified health condition, a previous COVID-19 infection, both, or none.

Data were summarized using counts with percentages for categorical variables and means with standard deviations for continuous variables. Variables were compared according to vaccination acceptance using chi-square tests for categorical data and correlation coefficients for continuous data. Fisher’s exact test was used for variables with cell counts <5. Data analysis was conducted in SPSS version 25, with *P* < .05 considered statistically significant.

## RESULTS

After excluding 22 cases with unknown vaccine acceptance status, the sample included 1,809 individuals. As depicted in Table I, most were White males. Additionally, most were members of the Army, and nearly 80% were members of Reserve or National Guard units. A small percentage included civilians who were contractors or employees of the Department of Defense. Generally, these individuals were relatively young adults, with a mean age of 32. BMI data were available for 1,793 individuals in the sample, and the group was largely comprised of overweight individuals, with a mean BMI of 27. Approximately 7% of the individuals in the sample had tested positive for COVID-19. In the overall sample, 61% accepted the vaccine, and 39% declined it. As shown in Table II, Black or African American personnel accepted the vaccine at lower rates than did White, Asian American, Hispanic, or American Indian personnel (*Χ*^2^(3) = 9.1, *P* = .03). Specifically, 54% of Black or African American personnel accepted the vaccine, whereas 61% and 67% of White personnel and personnel belonging to other racial groups agreed to be vaccinated, respectively.

**TABLE I.** Composition and COVID-19 Vaccine Acceptance Status of the Sample (*N* = 1,809)

Variable	Sample distribution (mean and SD or count and column %)
Age	32.2 (11.0)^a^
Body mass index	27.3 (3.8)
Race	196 (10.8%) Black or African American 344 (19.0%) Other 1,203 (66.5%) White 66 (3.6%) Declined to answer
Sex	382 (21.1%) Female 1,427 (78.9%) Male
Component	201 (11.1%) Active duty 482 (26.6%) National Guard 960 (53.1%) Reserve 164 (9.0%) Civilian 2 (0.1%) Declined to answer
Branch	1,305 (72.1%) Army 185 (10.2%) Navy 117 (6.5%) Coast Guard 44 (2.4%) Air Force 2 (0.1%) Marine Corps
Health Status	1,544 (85.4%) No health conditions 125 (6.9%) COVID-19 positive 128 (7.1%) Qualified health condition 11 (0.6%) COVID-19 and qualified health condition 1 (0.1%) No information available
COVID-19 vaccine acceptance^b^	699 (38.6%) Declined 1,110 (61.4%) Accepted

a Range: 19–76.

b Includes recovery from infection at least 28 days prior to vaccine being offered.

**TABLE II.** Comparison of Consent Rates by Categorical Study Variables (*N* = 1,809)

Variable	Accepted COVID-19 vaccine (*n* [row %])	*P*-value^a^
Race		.029
Black or African American	106 (54.1%)	
Other	230 (66.9%)	
White	732 (60.8%)	
Declined to answer	42 (63.6%)	
Sex		.309
Female	243 (63.6%)	
Male	867 (60.8%)	
Component		<.001
Active duty	133 (65.7%)	
Reserve or National Guard	854 (59.2%)	
Civilian	123 (75%)	
Branch		<.001
Army	740 (57.1%)	
Navy	146 (78.9%)	
Coast Guard	67 (57.3%)	
Air Force	32 (72.7%)	
Marine Corps	2 (100%)	
Civilian	123 (75%)	
Health status		.577
Qualified health condition, not COVID-19 positive	84 (65.6%)	
No qualified health condition, COVID-19 positive	71 (56.8%)	
Qualified health condition and COVID-19 positive	6 (54.5%)	
None	948 (61.4%)	
Unknown	1 (100%)	

a Chi-square test comparing consent status across all categories within the variable. Fisher’s exact test used for variables with cell counts <5.

Consent rates also varied according to military status. Members of Reserve or National Guard units were significantly less likely to accept the vaccine than active duty or civilian counterparts (*Χ*^2^(2) = 17.2, *P* < .001). In particular, 59% of individuals in Reserve or National Guard units accepted the vaccine, compared to 66% of active duty service members and 75% of civilians. Additionally, members of the Army and Coast Guard were less likely to consent to the vaccine than members of the Navy, Air Force, Marine Corps, or civilian employees (Fisher’s exact test *P* < .001). Specifically, among the Army and Coast Guard units, 57% of service members agreed to receive the vaccine, compared to 79% of Sailors, 73% of Airmen, 75% of civilians, and 100% of Marines. In other analyses summarized in Table II, we found that consent to receive the vaccine did not differ according to sex, prior COVID-19 exposure, or qualified health conditions. Point biserial correlations of continuous independent variables revealed that choice to accept the vaccine correlated with higher BMI (*r* = 0.07, *P* < .01) and older age (*r* = 0.16, *P* < .001). Although statistically significant, these correlations are small and suggest negligible relationships between these variables.

Because the rate of consent among Black or African American personnel differed from other races, follow-up analyses were conducted to determine whether race was confounded with other variables. One-way analyses of variance were computed to determine whether age or BMI varied between races. Age (*F*(3, 1,789) = 4.14, *P* < .01, η^2^= 0.007) and BMI (*F*(3, 1,789) = 4.51, *P* < .01, η^2^= 0.008) achieved significance. Black or African American, White, and Other groups did not differ from one another, whereas the Declined to Say group was older by approximately 4 years. Regarding BMI, Black or African American personnel (*M* = 28.1, standard deviation [SD] = 4.1) had slightly higher values than White (*M* = 27.2, SD = 3.7) or Other (*M* = 27.0, SD = 3.7) personnel, and they did not differ from those who Declined to Say their race (*M* = 27.3, SD = 3.8). Although these differences are statistically significant, they are unlikely to be meaningful as demonstrated by the small effect size differences among groups. Race differences emerged in regard to qualified health conditions and COVID status (*Χ*^2^(12) = 45.4, *P* < .001). Black or African American personnel were more likely to have a qualified health condition than the other groups. In particular, although comprising 10.8% of the sample, Black or African American personnel comprised 19.5% of those with a qualified health condition. Because of the race group differences in age, BMI, and health status, correlations between consent to receive the COVID vaccine and these variables were recomputed partialing out the effect of race. After controlling for race, consent choice correlated with age (*r* = 0.15, *P* < .001) and BMI (*r* = 0.08, *P* = .001). These values are essentially unchanged from the zero-order correlations, suggesting a negligible effect of race. Health status did not achieve a significant relationship with consent choice despite controlling for race (*r* = 0.007, *P* > 0.05).

## DISCUSSION

The emergence of new variants of the SARS-CoV-2 virus has lent new urgency to vaccinating populations against COVID-19.^9^ In military forces that do not mandate vaccination, hesitancy or refusal of the vaccine pose a threat to force readiness.^5^ We analyzed COVID-19 vaccine consent among deployed personnel in a joint environment as of May 2021 after emergency use authorization was granted for vaccine administration but before the announcement in August 2021 that vaccinations would become mandatory for service members.^10^ These data revealed that the majority of personnel consented to receive the vaccine, with no differences found according to prior COVID-19 infection. However, our analysis identified significant disparities in vaccine acceptance by race, military status, and service branch. Because these disparities may hinder attainment of vaccination levels necessary for herd immunity,^11^ targeted education initiatives are needed to overcome barriers to vaccine acceptance in the armed forces. Additionally, we identified a lower rate of vaccine acceptance among reserve and national guard personnel, suggesting a need for innovative approaches for educating these forces in the premobilization phase of deployment.

In a previous analysis of vaccination rates in the active component U.S. military, Lang and colleagues reported that by March 31, 2021, 27.2% of service members received at least one dose of the vaccine.^5^ In our analysis, 61% of the overall sample and 66% of active duty service members consented to receive the vaccine. Although our data reflect rapid uptake of COVID-19 vaccines since emergency use authorization was granted in December 2020, a large minority of personnel remained unvaccinated as of May 2021, with persistent disparities by race. In our sample, the vaccination rate was 7 percentage points lower among Black or African American than White personnel similar to the 10 percentage point difference reported in the earlier analysis by Lang et al.^5^ Whereas our analysis spanned a longer period since vaccine authorization as compared to Lang et al., there were also some demographic differences between the 2 samples. For example, Black or African American personnel accounted for 11% of our sample, compared to 16% of the active component U.S. Military.^5^

In the overall U.S. adult population, survey studies have identified greater reluctance to be vaccinated against COVID-19 among Black or African American adults compared to adults from other racial groups.^12,13^ This distrust may be related to the persistence of systemic racism in health care and requires targeted interventions to address inequities in vaccination uptake.^14,15^ Particularly, it is important to ensure that all service members have sufficient opportunities to have a frank and ongoing discussion with health care providers to address concerns and apprehensions related to vaccination. Additionally, research in the general population has identified a range of individual reasons for COVID-19 vaccine hesitancy or refusal, such as exposure to negative messages about the vaccine from family and friends and psychological reaction to a perceived loss of freedom.^16,17^

Observed discrepancies between the branches are worthy of consideration. Members of the Navy were more likely to accept the vaccine than those in the Army or Coast Guard. This may be attributable to the occupational specialties of Sailors in this sample, most of whom were assigned to a medical element. Arguably, their medical role and enhanced familiarity with vaccines may have contributed to their greater rates of acceptance. In the U.S., 52% of frontline health care workers in the United States had received the COVID-19 vaccine by March 2021,^18^ whereas in the general population, this milestone was not reached until June 2021.^19^ Reserve and national guard personnel were also less likely to accept the vaccine. We found no evidence that this difference was related to recovering from a prior COVID-19 infection, but it may be related to differences in perceptions of risk or information received about the vaccine compared to active duty personnel.

Several reports have described interventions to increase COVID-19 vaccine uptake among military personnel. Segal and colleagues described a series of initiatives in the Israeli Defense Forces (IDF) to foster vaccine uptake, including early vaccination of commanding officers and elite military units to set an example, sharing reliable information on the vaccine via social media, holding group counseling sessions led by medical officers, personally meeting with personnel who refused vaccination, requiring that personnel refusing vaccination accompany other service members to the vaccination sites, and requiring a unit-wide vaccination rate of 85% to ease restrictions on social activities and spaces.^4^ Talmy and colleagues also reported that individual consultation with a primary care physician increased vaccine uptake rates beyond unit-wide interventions in a single IDF unit.^20^ Many of these approaches were used by the command from which our data was drawn and may validate this approach as vaccine uptake was rapid.

Our analysis is subject to several limitations, including the single-site design and the retrospective nature of data collection for demographic, health, and vaccination data. These data points were collected from the EMR and were not externally validated. Additional data entry after the conclusion of the analysis period would have likely revealed higher rates of vaccine acceptance. Detailed information on reasons for vaccine refusal was not available but may have helped differentiate how many of the individuals declining vaccination were potentially amenable to agree to being vaccinated in the future. Analyzed deployed personnel primarily included members of the Army and Reserve/National Guard, so results for the overall sample may not be generalizable to other service branches and components. Additionally, information on military rank was not available for the present analysis.

In May 2021, the Department of Defense identified vaccine accessibility, education, personnel policies, and individual-level engagement using trusted messengers as key strategies for increasing acceptance of COVID-19 vaccines.^21^ Our analysis demonstrated that, as of May 2021, overall vaccine acceptance was greater among deployed military personnel than that reported in the U.S. population as a whole. However, Black or African American personnel had higher rates of refusal than White, Asian American, Hispanic, or American Indian personnel in this military sample. Further, it is not surprising that the soldiers of the National Guard and reserve components had higher rates of refusal given their straddling of the civilian and military worlds, which requires military leaders to develop unique methods for educating these populations to ensure optimal vaccine acceptance.
